# Development of a measure of genome sequencing knowledge for young people: The kids‐KOGS

**DOI:** 10.1111/cge.13607

**Published:** 2019-07-30

**Authors:** Celine Lewis, Bao S. Loe, Chris Sidey‐Gibbons, Christine Patch, Lyn S. Chitty, Saskia C. Sanderson

**Affiliations:** ^1^ North East Thames Regional Genetics Services Great Ormond Street Hospital NHS Foundation Trust London UK; ^2^ UCL Great Ormond Street Institute of Child Health London UK; ^3^ The Psychometrics Centre University of Cambridge Cambridge UK; ^4^ Patient Reported Outcomes, Value and Experience (PROVE) Centre Brigham and Women's Hospital Boston Massachusetts; ^5^ Genomics England Queen Mary University of London London UK; ^6^ Counselling, Society and Ethics Research Wellcome Genome Campus Cambridge UK; ^7^ Florence Nightingale Faculty of Nursing and Midwifery King's College London London UK; ^8^ Institute of Health Informatics University College London London UK

**Keywords:** item response theory, measurement instrument, patient education, patient knowledge, whole genome sequencing, young people

## Abstract

Genome sequencing (GS) is increasingly being used to diagnose rare diseases in paediatric patients; however, no measures exist to evaluate their knowledge of this technology. We aimed to develop a robust measure of knowledge of GS (the kids‐KOGS') suitable for use in the paediatric setting as well as for general public education. The target age was 11 to 15 year olds. An iterative process involving six sequential stages was conducted to develop a set of draft true/false items. These were then administered to 539 target‐age school pupils (mean 12.8; SD ± 1.3), from the United Kingdom. Item‐response theory was used to confirm the psychometric suitability of the candidate items. None of the Items was identified as misfits. All 10 items performed well under the two‐parameter logistic model. The internal consistency of the test was 0.84 (Cronbach alpha value) indicating excellent reliability. The mean kids‐KOGS score in the sample overall was 4.24 (SD; 2.49), where 0 = low knowledge and 10 = high knowledge. Age was positively associated with score in a multivariate linear regression. The kids‐KOGS is a short and reliable tool that can be used by researchers and healthcare professionals offering GS to paediatric patients. Further validation in a clinical setting is required.

## Graphical abstract

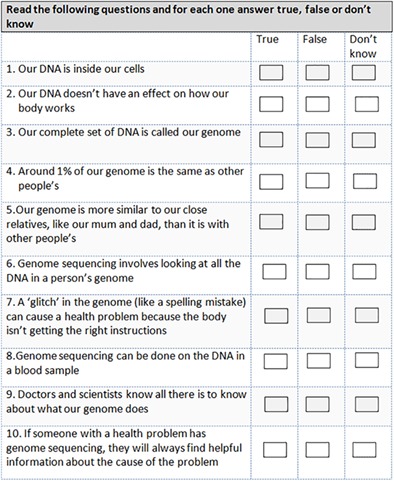

## 1 INTRODUCTION

In 2019, genome sequencing (GS) will become part of the NHS England commissioned national genomic medicine service for rare disease and cancer, facilitating systematic access to genomic testing across the country.^1^ This service follows from the 100 000 Genomes Project, the largest national sequencing project of its kind in the world delivering research on how best to use genomics in healthcare and interpret data to help patients.^2^

Children and young people are significant benefactors of GS technology: of the rare disease proband participants in the 100 000 Genomes Project, around a quarter of them were 15 years of age or under at the time of taking part (data accessed from the Genomics England Research Environment, 11th November, 2018). GS has been shown to improve diagnostic yield over targeted gene sequencing in the paediatric setting as the reported diagnostic yield in previously unsolved paediatric cases is already around 40% and will likely continue to increase as knowledge grows.^3^ This offers an end to the “diagnostic odyssey” for many children (and their parents) with rare diseases.^4^ Other benefits include enabling targeted therapy for some, reproductive planning and opportunities to make contact with other families whose children have similar conditions.^5^

A key challenge in implementing GS in clinical practice is how to counsel patients so that they can make an informed choice, defined as a decision “that is based on relevant knowledge, consistent with the decision‐maker's values and behaviourally implemented”.^6^ With regards to knowledge, in the context of GS this will require health professionals to explain to patients (including parents and their children) about issues including what GS is, the possible genomic results that may be revealed and the limitations and uncertainties.^7^

In 2018, our group developed the knowledge of GS (KOGS) measure, to address the need for a valid, reliable measure that can be used with individuals in a range of settings.^8^ The measure is “context‐neutral,” that is, the items can be administered to patients or other stakeholders (eg, healthcare providers, students, general public) regardless of the clinical or other context. This measure was developed with and administered to adults aged 18 years and over.

Engaging young people as active participants in the decision‐making process is increasingly seen as good clinical practice^9^ and the few qualitative studies about GS that have been conducted with this age group suggest that they want to be engaged in that process.^10^, ^11^, ^12^ In the 100 000 Genomes Project, 11 to 15 year olds invited to take part in the study were given tailored participant information sheets, encouraged to be active participants in the decision‐making process, and if they wanted to take part, sign an “assent” form, in addition to their parents ultimately consenting on their behalf.^13^ (Patients aged 16 years and upwards were, in contrast, considered adults and consented on their own behalf). Currently, no knowledge measure exists that is specifically aimed at young people who may be involved in decision‐making about GS. Given that they make up a significant proportion of patients who are likely to benefit from GS in the new Genomic Medicine Service, measures to evaluate their understanding of the technology (purpose, benefits, limitations, etc.) are important. We therefore built on our earlier work on the KOGS to develop a new measure which we have called the kids‐KOGS, to address this unmet need.

## 2 MATERIALS AND METHODS

### 2.1 Ethics and consent

Approval for this study was obtained from the NHS Research Ethics Committee West Midlands (15/WM/0258) and from each of the schools involved. Participation in the study was voluntary and consent to take part was implied by the questionnaire being completed and returned.

### 2.2 Target age groups

The measure was developed for a target age group of 11 to 15 year olds.

### 2.3 Selection of knowledge domains

During the development of the adult KOGS,^8^ three “context‐neutral” knowledge domains were identified: (a) what is a genome, (b) what is involved in having GS performed, and (c) the limitations and uncertainties of GS. We therefore focused on these three domains for the new kids‐KOGS measure so that it could similarly be used in a range of clinical and other contexts.

### 2.4 Item development

A set of 10 true/false knowledge items (Table 1) were developed to cover each of the three domains, which were informed via *six* sequential phases.

**Table 1:** The final 10‐item knowledge of genome sequencing measure for young people (kids‐KOGS)

Read the following questions and for each one answer true, false or don't know			
	True	False	Don't know
1. Our DNA is inside our cells	□	□	□
2. Our DNA doesn't have an effect on how our body works	□	□	□
3. Our complete set of DNA is called our genome	□	□	□
4. Around 1% of our genome is the same as other people's	□	□	□
5. Our genome is more similar to our close relatives, like our mum and dad, than it is with other people's	□	□	□
6. Genome sequencing involves looking at all the DNA in a person's genome	□	□	□
7. A ‘glitch’ in the genome (like a spelling mistake) can cause a health problem because the body isn't getting the right instructions	□	□	□
8. Genome sequencing can be done on the DNA in a blood sample	□	□	□
9. Doctors and scientists know all there is to know about what our genome does	□	□	□
10. If someone with a health problem has genome sequencing, they will always find helpful information about the cause of the problem	□	□	□

The *first phase* involved speaking directly with young people aged 11 to 15 years to identify the key questions they would want answered if they were considering having GS. To do this, CL conducted 16 semi‐structured qualitative interviews with 11 to 15 year olds taking part in the 100 000 Genomes Project (manuscript in preparation) and CL and SS conducted two school visits in London (one primary school and one secondary school). To ensure that the pupils had some background understanding of genomics, they were first shown a short section of an educational video.^14^ Ten broad questions were identified in this phase which covered the three domains, including “What is a genome?”, “How do you do genome sequencing?”, “How accurate are the results from genome sequencing?” and “Will you always get an answer from genome sequencing?” (see: Table S1).

The *second phase* was to map items from an early 17‐item draft of the (adult) KOGS measure^8^ (including both true and false items), onto the questions that had been identified by young people for potential inclusion in the kids‐KOGS (see Table 1). After this exercise, three questions remained that had not been addressed using the draft 17‐item (adult) KOGS; “What is DNA?”, “How does our genome affect our health?” and “How similar is our genome to other people's?” Three items were developed specifically to address these questions.

The *third phase* was to develop a true and false version of each of the items. These were either taken from the previously developed true and false items from the 17‐item draft KOGS or developed specifically for the kids‐KOGS by CL and SS. This resulted in 20 items (10 true and 10 false) (see Table S2). In the *fourth phase*, we randomly chose five numbers between 1 and 10 using a random number generator, and the true versions of these items were selected, resulting in 5 true and 5 false items. In the *fifth phase*, cognitive interviews were conducted with two young people taking part in the 100 000 Genomes Project (who had taken part in the qualitative interviews), two science teachers and one adult parent to provide feedback on wording and comprehension. Minor changes to wording were made at this stage. In the final *sixth phase*, the 10 items were then administered to a group of 83 pupils at a school in the East of England aged 11 to 12 and feedback was sought on the wording and comprehension. At this stage, one of the false items was swapped for a true item as it was considered by pupils to be ambiguous (“whole genome sequencing is done through an X‐ray”) leaving 6 true and 4 false items in the knowledge scale.

### 2.5 Questionnaire administration

The 10‐item knowledge scale (Table 1) was administered in‐person to 554 students across six schools (four secondary schools and two primary schools) in London, Essex, and Wiltshire between July 2017 and July 2018.

### 2.6 Psychometric and statistical analyses

#### 2.6.1 Factor analysis

Preliminary analysis confirmed the suitability of the data for conducting a factor analysis to ensure that the 10 items measure a single construct (Bartlett's test of sphericity: X^2^ = 932.38, df = 45, *P* < 0.001, KMO = 0.86). A maximum likelihood estimator with tetrachoric correlation was conducted using the “Psych” package for the R Statistical Programming Environment.^15^ Both parallel and scree plot analysis were used to identify the number of factors in the data.

#### 2.6.2 Psychometric analyses

We used item response theory (IRT) to analyse the psychometric properties of the 10‐item scale. IRT is widely used to evaluate the relationship between the test takers' ability (in this case, knowledge of genome sequencing) and their responses to individual questions assessing knowledge of genome sequencing.^16^ IRT is increasingly favoured as a psychometric analysis tool as it offers deeper insights into the way in which questionnaires function. Under IRT paradigms, further investigation in psychometric performance is possible using sophisticated methods (eg, differential item functioning, local dependency, person and item fit). We employed the two‐parameter logistic (2PL) IRT model, which is often applied to scales with dichotomous (ie, yes/no) responses, for item analyses, including item difficulty and discrimination parameters.^17^

We assessed the quality of the nascent scale by evaluating the fit of the data to the model at the item, person, and whole scale levels. Additionally, we ensured that the data did not violate the assumptions of the model, namely; dimensionality (assessed using factor analysis described above), local independence of items, and differential item functioning. Further details of these analyses are provided in Supporting Information: Methods section.

Participants were split into two groups based on the median age (13 years). Those who were younger (n = 231) than the median group were placed in the first group while those who were older (n = 279) than the median group were placed in the second group. In the sex group, 202 males were placed in group 1 while 307 females were placed in group 2. One participant chose not to reveal his/her sex and was thus excluded from the differential item functioning (DIF) analysis.

The 2PL model, overall model fit, person, item fit and DIF analysis were all estimated in the R programming environment (R Development Core Team, 2018), with the multidimensional IRT (MIRT) package^18^ and the lordif package.^19^

### 2.7 Statistical analyses

The proportion of “correct” responses for each of the 10 items was described. Bivariate analysis was conducted to examine whether there were differences in responses to the total kids‐KOGS score according to age, sex and school. We used a *t* test to examine the association between the Kids‐KOGS score and age, a Pearson's correlation for age and an analysis of variance (ANOVA) for school. A multivariate linear regression was conducted to explore the independent associations between the dependent and three independent variables. All tests were two‐tailed and significance level was set at *P* < .05. Statistical analyses were conducted using SPSS v22.

## 3 RESULTS

### 3.1 Sample characteristics

In total, 554 participants were offered and attempted the questionnaire (Table 2). Fifteen participants did not fully complete the questionnaire. Thus, the data of 539 participants were included for analysis. More respondents were female (59.2%) than male (40.4%), and the mean age was 12.8 (±1.3) years.

**Table 2:** Participant characteristics

Characteristic	% (n)
Age, years	
Mean (SD)	12.8 (±1.3)
11	17.3% (93)
12	30.2% (163)
13	18.7% (101)
14	19.9% (107)
15	13.9% (75)
Sex	
Female	59.2% (319)
Male	40.4% (218)
Missing	0.2% (1)
School	
Primary 1	6.7% (36)
Primary 2	3.5% (19)
Secondary 1	26.0% (140)
Secondary 2	25.0% (135)
Secondary 3	18.2% (98)
Secondary 4	20.6% (111)

### 3.2 Psychometrics

**Figure 1:**
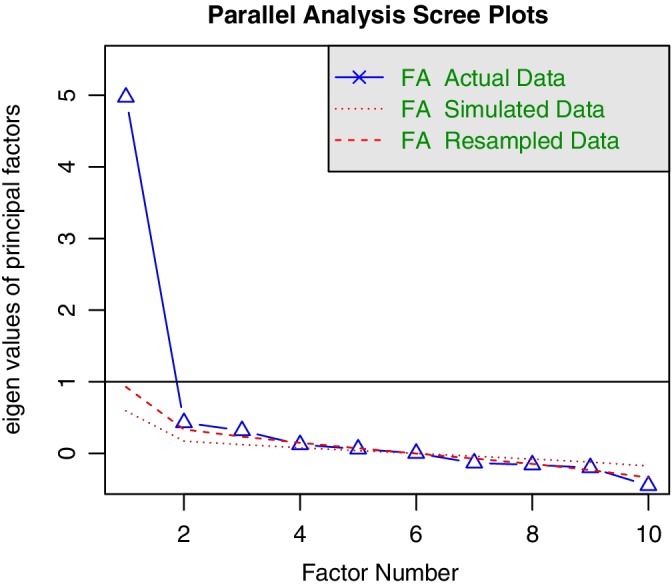
Parallel analysis scree plots [Colour figure can be viewed at https://wileyonlinelibrary.com]

Scree‐plot analysis confirmed that the scale does assess a single underlying construct (ie, knowledge of genome sequencing, see Figure 1). The eigenvalue of the first factor was 4.97, with no other factor greater than 1. Monte‐Carlo analysis suggested that some mild multidimensionality was present, but the additional factors did not account for much variance and may have been spurious, given the marginality of the result and known issues with this procedure.^20^, ^21^, ^22^ Factor loadings were all greater than 0.30, ranging between 0.35 and 0.66 (see Table S3). It was, therefore, deemed appropriate to treat the scale as a unidimensional measure and continue with IRT analyses on the 10 items.

**Figure 2:**
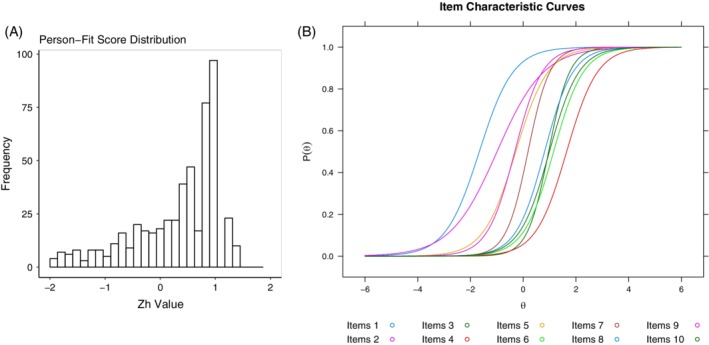
Psychometric properties of the knowledge of genome sequencing measure for young people (kids‐KOGS) [Colour figure can be viewed at https://wileyonlinelibrary.com]

We fitted data from 539 respondents to the 2PL IRT. Twenty‐nine participants were removed from further analysis because of aberrant responses patterns (eg, correct responses to hard questions, incorrect response to easy questions; ZH value | > ±2|). We refitted the 2PL model using data from the remaining 510 participants and both items and person fitted the model (see Figure 2A and Table 3). Local dependency was not evident for any items.

**Table 3:** Item fit statistics

Items	S‐X^2^	Df	*P*‐value
1	8.26	4	.08
2	9.18	6	.16
3	6.59	5	.25
4	7.11	5	.21
5	6.14	6	.41
6	5.05	6	.54
7	5.31	5	.38
8	6.55	6	.36
9	4.91	5	.43
10	8.17	6	.23

Abbreviation: Df, Degrees of freedom.

The item difficulty and discrimination parameter estimates for the final 10 items are shown in Table 4. Item 1 is the easiest item with a difficulty (*θ*) estimate of −1.66, while item 4 is the hardest with a difficulty (*θ*) estimate of 1.65. All items have acceptable discrimination values ranging from 1.13 (item 2) to 2.51 (item 3). The item characteristic curves for each item is displayed in Figure 2B. No items displayed DIF for either age or sex, indicating that the scale functions uniformly across demographic groups. Items were flagged as displaying DIF (Table S4A,B), indicating that respondents with the same underlying true ability from different sub‐sample groups (sex and age) did not have a different probability of endorsing the same response.

**Table 4:** Item parameter estimates

Items	Discrimination (a)	SE	Difficulty (b)	SE
1	1.56	0.24	−1.66	0.18
2	1.13	0.16	−0.99	0.15
3	2.51	0.37	0.97	0.09
4	1.71	0.27	1.65	0.17
5	1.64	0.21	−0.28	0.08
6	1.77	0.25	1.15	0.12
7	2.42	0.32	0.18	0.07
8	1.85	0.25	0.81	0.10
9	1.92	0.25	−0.32	0.08
10	1.79	0.242	0.99	0.107

With reference to the model fit, the M_2_ statistic was 73.01 (35) with a *P*‐value <.01. The Root mean square error of approximation (RMSEA) was 0.05 (95% CI 0.03, 0.06), standardised root mean square residual was 0.04, and the comparative indices (comparative fit index at 0.98 and tucker lewis index at 0.97) were within the recommended cut‐off criteria. Collectively, the result of the fit indices indicates an acceptable model fit. The internal consistency of the test was 0.84 after converting the peak of the test information to the equivalent of a Cronbach alpha value (Figure S1A,B).

### 3.3 Descriptive analyses

As shown in Table 5, the item that was most frequently answered correctly was “Our DNA is inside our cells” (true, 83.1% correct) followed by “Our DNA doesn't have an effect on how our body works” (false, 69.2% correct). The items that were least often answered correctly were “Around 1% of our genome is the same as other people's” (false, 14.1% correct) and “Our complete set of DNA is called our genome” (true, 21.9% correct).

**Table 5:** Proportion of correct responses to each of the 10 knowledge of genome sequencing measure for young people (kids‐KOGS) items

Item	Correct	Incorrect	Don't know
Item 1: Our DNA is inside our cells (True)	83.1% (448)	3.2% (17)	13.7% (74)
Item 2: Our DNA doesn't have an effect on how our body works (False)	69.2% (373)	12.6% (68)	18.2% (98)
Item 9: Doctors and scientists know all there is to know about what our genome does (False)	59.0% (318)	4.5% (24)	36.5% (197)
Item 5: Our genome is more similar to our close relatives, like our mum and dad, than it is with other people's (True)	58.6% (316)	2.0% (11)	39.3% (212)
Item 7: A “glitch” in the genome (like a spelling mistake) can cause a health problem because the body isn't getting the right instructions (True)	45.1% (243)	3.9% (21)	51.0% (275)
Item 8: Genome sequencing can be done on the DNA in a blood sample (True)	29.1% (157)	2.2% (12)	68.6% (370)
Item 10: If someone with a health problem has genome sequencing, they will always find helpful information about the cause of the problem (False)	24.1% (130)	14.5% (78)	61.2% (330)
Item 6: Genome sequencing involves looking at all the DNA in a person's genome (True)	22.3% (120)	8.5% (46)	69.2% (373)
Item 3: Our complete set of DNA is called our genome (True)	21.9% (118)	3.3% (18)	74.6% (402)
Item 4: Around 1% of our genome is the same as other people's (False)	14.1% (76)	9.3% (50)	76.6% (413)

### 3.4 Statistical analyses

The mean (SD) kids‐KOGS score in the sample overall was 4.24 (2.49), where 0 = low knowledge and 10 = high knowledge. There were differences by sex, age, and school in bivariate analyses. The mean kids‐KOGS score was higher among girls than boys (4.44 vs 4.09, respectively, *t* [535] = 1.61, *P* = .0002), and higher among older children (*r* = .25, n = 539, *P* = 6.77 x 10^−9^). When broken down by age, the mean scores and ranges were as follows: 3.29; 0 to 8 (11 years); 4.09; 0 to 10 (12 years); 3.96; 0 to 10 (13 years); 4.68; 0 to 10 (14 years); 5.47; 0 to 10 (15 years). There was also a significant difference between the six schools ([F95, 522] = 11.51, *P* = 1.42^−10^), with the mean kids‐KOGS score higher across secondary schools than primary schools (4.43 vs 2.54, respectively, *t*[552] = 5.60, *P* = .009). When sex, age and (the six) schools were entered into a multivariate linear regression, only age remained independently associated with knowledge (*t* = 6.32, *P* = 5.59 x 10^−10^).

## 4 DISCUSSION

This is the first measure of KOGS that has been developed specifically for young people. The strengths of the methodological approach are that (a) the questions were developed with young people (including those with rare diseases) to ensure they addressed aspects of GS they thought were important, (b) feedback on wording was sought with a range of stakeholders including young people at multiple stages, (c) analysis of dimensionality and item properties was conducted using a rigorous psychometric measure development approach, and (d) the measure can be used in a range of settings including with paediatric patients in clinic as well as with young people in schools.

As GS becomes mainstreamed into clinical care to diagnose young people with rare diseases, it will be increasingly important to assess their understanding of genetics and genomics as well as the limitations and uncertainties of the technology. This is particularly important given that research has shown that while this group is interested in and willing to undertake genetic testing, they have concerns about the implications of testing and the potential risks and limitations.^11^, ^23^ An important limitation of the technology is that currently around 60% of paediatric patients do not get a diagnosis from GS^3^ which may lead to feelings of frustration and disappointment.^24^, ^25^ Studies have also shown that age may play a factor in young people's understanding of genetics^26^ as well as whether there is a family history of a genetic condition.^27^ Using this measure, health professionals may be able to identify those young people that might have limited knowledge or misunderstandings about WGS and who may therefore require more in‐depth counselling or information provision.

We found age to be significantly associated with knowledge: older pupils scoring higher on the kids‐KOGS than younger pupils. This finding differs to that of Sabatello et al.^28^ who did not find any differences in objective genomic knowledge between 14 and 17 year olds. However, their study included a more limited age range and a different set of questions. Our findings might reflect the National Curriculum in England where concepts, such as genetics and DNA are only formally introduced into science lessons at Key Stage 3 which is during the first 3 years of secondary school (ages 11‐14) and genomics at general certificate of secondary education (GCSE) level (ages 15‐16).^29^ The term “genome” is also a more recent concept and may therefore be less well understood within the public sphere. A recent report on genome editing found that there was confusion about the term ‘genome’ even among patients and families affected by rare diseases who might be considered likely to encounter the term.^30^ Introduction to the concept of DNA from the age of 11 might also explain why the two questions most frequently answered correctly related to the location and function of DNA rather than questions related to genomics which is only formally introduced as school at GCSE level.

The main limitation of this study is that the sample was not evenly balanced between male and female participants, however, sex was not found to be significant in the linear regression. We have also not yet had the opportunity to use the kids‐KOGS in a clinic setting with young people. Another limitation is that a nested structure may exist because the scales were administered to students from difference schools, indicating possible dependencies in the data structure. Hence, future research is warranted to investigate the possibility of multilevel dependencies between schools in greater detail.

In conclusion, we have used a rigorous approach to develop a brief, reliable measure of knowledge of GS for young people which can be used in a range of settings including the paediatric clinic as well as with young people in schools. Future research could focus on using the measure to evaluate how effective pre‐test counselling appointments are at explaining GS as well as to evaluate interventions aimed at young people such as online educational resources.

## CONFLICT OF INTEREST

Nothing to declare.

## Supporting information


**Appendix S1** Supporting information

## Data Availability

An SPSS file of the total dataset is available on request to the corresponding author.
