# Supplementary material for: Development of a measure of genome sequencing knowledge for young people: The kids‐KOGS
Source: Clin Genet. 2019 Jul 30;96(5):411–7. doi: 10.1111/cge.13607 (PMC6851564; doi:10.1111/cge.13607)
Supplement: Supplementary file 1 — Appendix S1 Supporting information [file CGE-96-411-s001.pdf]

Supporting Information: Methods

Both person and item fit to the 2PL model were evaluated. Person fit validates the extent to which the trait level estimates are accurate based on the participants' aggregated responses across items.[1] Fit analysis can be used to detect and remove aberrant response patterns to create a more accurate assessment tool.[2] The person fit statistics in this research is based on the Zh index.[3] An arbitrary Zh-value of greater than |± 2| was considered reflective of "atypical" response patterns that warranted further inspection.[4] Item fit, on the other hand, investigates the validity of the item parameters.[1] The S-X^2 likelihood based goodness of fit index was used for detecting item misfit.[5] Items with significant differences of *p-value* < 0.01 were considered to misfit the model and were removed from further analysis.

We conducted local dependency analysis to ensure that items were not locally dependent on each other. The Yen's Q3 method of correlated residuals [6] was used to identify local dependency. Items with residual correlations above .20 were suspected of local dependency and thus removed from further analysis.

In terms of model fit, the M_2 statistics [7] was selected as a goodness-of-fit assessment to identify poor fitting models, along with four additional fit indices, the comparative fit index (CFI), the Tucker-Lewis index (TLI), the root-mean-square error of approximation (RMSEA) and the standardised root mean square residual (SRMR). The cut-off criteria for these indices follow those recommended by Hu & Bentler [8] and Maydeu-Olivares and Joe.[9] The recommended cut-off for these fit indices indicates an adequate fit between the hypothesised model and the observed data.

Differential Item Functioning (DIF) analysis was also conducted to assess item variance for age and sexgender. DIF analysis is used to detect item bias and could indicate that an item may have different probabilities of success across different sub-sample groups after controlling for the underlying trait measured by the test.[10] DIF was accessed using an ordinal regression framework.[11] In

For Review Only

these analyses, the dependent variable was the item responses (TRUE/FALSE), and the predictor was the level of the ability being measured. The base model (model 1) uses only the level of ability as a predictor of participants' responses. The second model (model 2) uses both level of theta and the grouping variable (i.e. age or sex~~gender~~) to predict responses. DIF is identified if items in model 2 predicts item responses statistically significantly better than model 1. A third model (model 3) includes an interaction term and tests whether the DIF is uniform (impact is consistent across ability level) or non-uniform (impact varies by ability level) between items. The changes in the McFadden's pseudo R-square between the models are used to detect DIF, with a difference of greater than .13 indicating that a meaningful DIF exists.[11]

For Review Only

References

1. Reise SP. A comparison of item-and person-fit methods of assessing model-data fit in IRT. *Applied Psychological Measurement.* 1990;14(2):127-137.
2. Tatsuoka KK, Tatsuoka MM. Detection of aberrant response patterns and their effect on dimensionality. *Journal of Educational Statistics.* 1982;7(3):215-231.
3. Drasgow F, Levine MV, Williams EA. Appropriateness measurement with polychotomous item response models and standardized indices. *British Journal of Mathematical and Statistical Psychology.* 1985;38(1):67-68.
4. Felt JM, Castaneda R, Tiemensma J, Depaoli S. Using Person Fit Statistics to Detect Outliers in Survey Research. *Frontiers in psychology.* 2017;8:863.
5. Orlando M, Thissen D. Likelihood-based item-fit indices for dichotomous item response theory models. *Applied Psychological Measurement.* 2000;24(1):50-64.
6. Yen WM. Scaling performance assessments: Strategies for managing local item dependence. . *Journal of educational measurement.* 1993;30(3):187-213.
7. Maydeu-Olivares A, Joe H. Limited information goodness-of-fit testing in multidimensional contingency tables. *Psychometrika.* 2006;71(4):713.
8. Hu LT, Bentler PM. Cutoff criteria for fit indexes in covariance structure analysis: Conventional criteria versus new alternatives *Structural equation modeling: a multidisciplinary journal.* 1999;6(1):1-55.
9. Maydeu-Olivares A, Joe H. Assessing approximate fit in categorical data analysis. *Multivariate Behavioral Research.* 2014;49(4):305-328.
10. Camilli G, Shepard LA, Shepard L. *Methods for identifying biased test items.* Vol Vol 4: Sage; 1994.
11. Swaminathan H, Rogers HJ. Detecting differential item functioning using logistic regression procedures. *Journal of educational measurement.* 1990;27(4):361-370.

Supporting information Table 1: Development of the kids-KOGS

Questions identified by young people related to 'what is a genome' 'what is genome sequencing' and 'limitations and uncertainties'	KOGS draft 17-item version	Items developed specifically for the kids-KOGS
**What is a genome?**		
What is DNA?		DNA is a molecule inside the nucleus of your body's cells (true)
What is a genome?	A person's genome is their complete set of DNA (true)<br><br>A person's genome is the complete set of cells in their body (false)	
What does our genome do?	A person's genome is their body's 'instruction manual' containing the information needed to make them, run them and repair them (true)	
How does our genome affect our health?		Changes in the genome can cause problems because the body isn't getting the correct instructions (true)
Is our DNA the same as our parents' DNA?	A person shares more of their DNA with their family members than they do with other people.	
Does everyone's genome look the same?		Over 99% of our genome is the same as other people's (true)
**What is genome sequencing?**		
What is genome sequencing?	Whole genome sequencing involves looking at most of the DNA in a genome (true)<br><br>Whole genome sequencing involves looking at around half of the DNA in a genome (false)	
How do you do genome sequencing?	Whole genome sequencing can be done on a blood sample (true)<br><br>Whole genome sequencing cannot be done on a sample from a simple blood test (false)	

For Review Only

Limitations and uncertainties		
How accurate are the results?	The effects of all DNA variants identified through whole genome sequencing on disease are known (false)<br><br>The effects of all DNA variants identified through whole genome sequencing on disease are not yet known (true)	
Are there any risks from genome sequencing?	Whole genome sequencing always provides a person with meaningful information about their health (false)<br><br>Whole genome sequencing may not provide a person with any meaningful information about their health (true).	

For Review Only

Table 2: True and False Items

Questions identified by young people related to 'what is a genome' 'what is genome sequencing' and 'limitations and uncertainties'	True	False
**What is a genome?**		
What is DNA?	DNA is a molecule inside the nucleus of your body's cells (true)	DNA is the complete set of cells in your body (false)
What is a genome?	A person's genome is their complete set of DNA (true)	A person's genome is the 1% of their DNA that makes proteins (false)
What does our genome do?	A person's genome is their body's 'instruction manual' containing the information needed to make them, run them and repair them (true)	A person's genome doesn't have any effect on how their body functions (false)
How does our genome affect our health?	Changes in the genome can cause problems because the body isn't getting the correct instructions (true)	Changes in the genome will always cause health problems (false)
Is our DNA the same as our parents DNA?	A person shares more of their DNA with their family members than they do with other people (true)	A person shares less of their DNA with family members than with other people (false)
Does everyone's genome look the same?	Over 99% of our genome is the same as other people's (true)	Around 1% of our genome is the same as other people's (false)
**What is genome sequencing?**		
What is genome sequencing?	Whole genome sequencing involves looking at most of the DNA in a genome (true)	Whole genome sequencing involves looking at around half of the DNA in a genome (false)
How do you do genome sequencing?	Whole-genome sequencing can be done on a blood sample (true)	Whole genome sequencing is done through an x-ray (false)
**Limitations and uncertainties**		
How accurate are the results?	The effects of all DNA variants identified through whole genome sequencing on disease are not yet known (true)	The effects of all DNA variants identified through whole genome sequencing on disease are known (false)
Are there any risks from genome sequencing?	Whole-genome sequencing may not provide a person with any meaningful information about their health (true)	Whole genome sequencing always provides a person with meaningful information about their health (false)

For Review Only

Table 3: Standardised Factor Loadings for a Single Factor

Index	Factor Loadings	Communality	Residual
1	0.35	0.13	0.87
2	0.39	0.15	0.85
3	0.60	0.36	0.64
4	0.43	0.19	0.81
5	0.53	0.29	0.71
6	0.52	0.27	0.73
7	0.66	0.43	0.57
8	0.56	0.32	0.68
9	0.58	0.33	0.67
10	0.53	0.29	0.71

For Review Only

Table 4a: DIF for Age Groups

item	ncat	Model 1 vs Model 2	Model 1 vs Model 3	Model 2 vs Model 3
1	2	0.014	0.016	0.002
2	2	0.031	0.031	0.000
3	2	0.001	0.003	0.002
4	2	0.003	0.007	0.004
5	2	0.018	0.018	0.000
6	2	0.000	0.001	0.000
7	2	0.001	0.004	0.003
8	2	0.009	0.009	0.001
9	2	0.003	0.005	0.002
10	2	0.000	0.001	0.001

ncat - Grouping variable. Model 1 vs Model 2 - The difference in McFadden's Pseudo R^2 between model 1 and model 2. Model 1 vs Model 3 - The difference in McFadden's Pseudo R^2 between model 1 and model 3.Model 2 vs Model 3 - The difference in McFadden's Pseudo R^2 between model 2 and model 3.

For Review Only

Table 4b: DIF for Gender Groups

item	ncat	Model 1 vs Model 2	Model 1 vs Model 3	Model 2 vs Model 3
1	2	0.003	0.006	0.003
2	2	0.000	0.001	0.000
3	2	0.001	0.002	0.002
4	2	0.007	0.012	0.005
5	2	0.001	0.011	0.010
6	2	0.013	0.013	0.000
7	2	0.005	0.009	0.004
8	2	0.003	0.007	0.004
9	2	0.000	0.004	0.004
10	2	0.000	0.000	0.000

ncat - Grouping variable. Model 1 vs Model 2 - The difference in McFadden's Pseudo R^2 between model 1 and model 2. Model 1 vs Model 3 - The difference in McFadden's Pseudo R^2 between model 1 and model 3.Model 2 vs Model 3 - The difference in McFadden's Pseudo R^2 between model 2 and model 3.

Supporting Information: Figures 1a and 1a

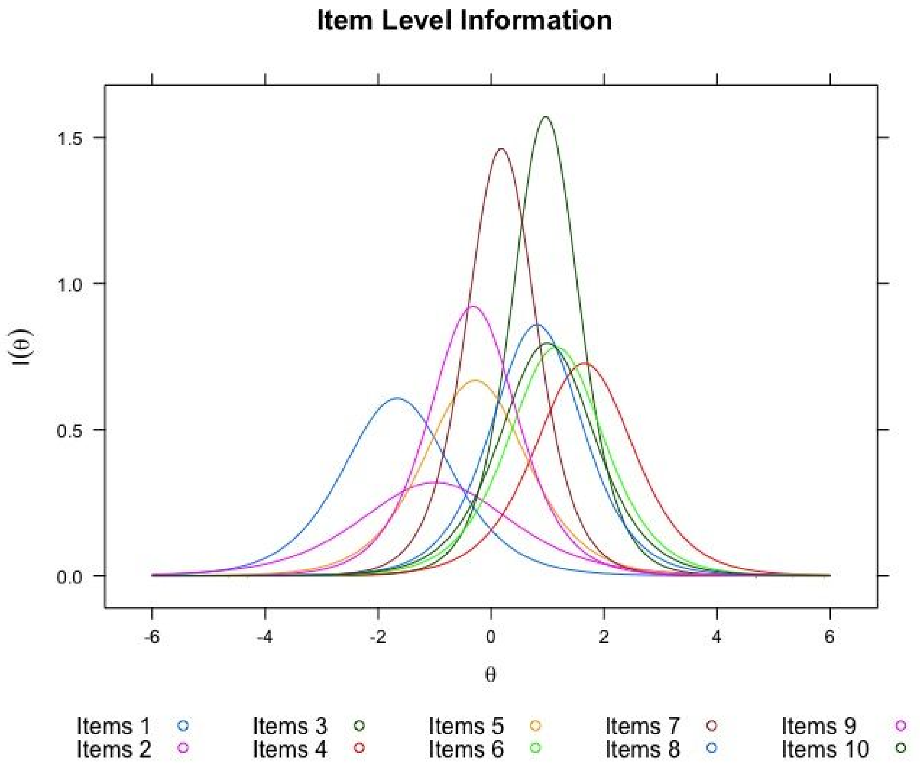

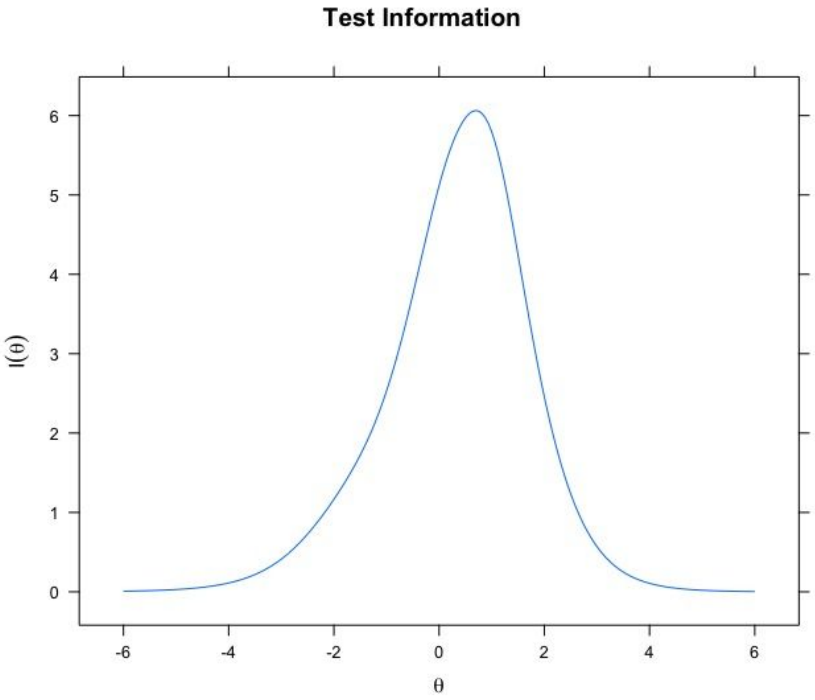

1a: Item level test information

1a: Overall test level information
